# Perceptions and expectations of health-related quality of life among geriatric patients seeking emergency care: a qualitative study

**DOI:** 10.1186/s12877-019-1228-6

**Published:** 2019-08-05

**Authors:** Scott M. Dresden, Danielle M. McCarthy, Kirsten G. Engel, D. Mark Courtney

**Affiliations:** Department of Emergency Medicine, Feinberg School of Medicine, Northwestern University, 211 E. Ontario St., Suite 200, Chicago, IL 60611 USA

**Keywords:** Emergency medicine, Geriatrics, Health related quality of life, Recovery of function, Anxiety, Qualitative research

## Abstract

**Background:**

Health-related quality of life (HRQoL), encompassing social, emotional, and physical wellbeing is an important clinical outcome of medical care, especially among geriatric patients. It is unclear which domains of HRQoL are most important to geriatric patients and which domains they hope to address when using the Emergency Department (ED). The objective of this study was to understand which aspects of HRQoL are most valued by geriatric patients in the ED and what expectations patients have for addressing or improving HRQoL during an ED visit.

**Methods:**

This was a qualitative focus group study of geriatric ED patients from an urban, academic ED in the United States with > 16,500 annual geriatric visits. Patients were eligible if they were age > =65 years and discharged from the ED within 45 days of recruitment. Semi-structured pilot interviews and focus groups were conducted several weeks after the ED visit. Participants shared their ED experiences and to discuss their perceptions of the subsequent impact on their quality of life, focusing on the domains of physical, mental, and social health. Latent content and constant comparative methods were used to code focus group transcripts and analyze for emergent themes.

**Results:**

Three individuals participated in pilot interviews and 31 participated in six focus groups. Twelve codes across five main themes relating to HRQoL were identified. Patients recalled: (1) A strong desire to regain physical function, and (2) anxiety elicited by the emotional experience of seeking care in the emergency department, due to uncertainty in diagnosis, treatment, and prognosis. In addition, patients noted both (3) *interpersonal* impacts of health on quality of life, primarily mediated primarily by social interaction, and (4) an *individual* experience of health and quality of life mediated primarily by mental health. Finally, (5) patients questioned if the ED was the right place to attempt to address HRQoL.

**Conclusions:**

Patients expressed anxiety around the time of their ED visit related to uncertainty, they desired functional recovery, and identified both interpersonal effects of health on quality of life mediated by social health, and an individual experience of health and quality of life mediated by mental health.

## Background

Geriatric patients (age 65 and older) use the emergency department (ED) more than any other age group [1]. However, EDs were not traditionally designed for older adults [2]. They are loud, chaotic, and care focuses on rapid triage and diagnosis of the acute issue, rather than addressing underlying issues such as multi-morbidity and polypharmacy which are common in geriatric patients [2]. To address this mismatch, Geriatric Emergency Department Innovations (GEDIs) have been developed to better care for older adults in the ED. [3] These GEDIs include dedicated geriatric emergency departments (GEDs) and innovations in ED processes such as ED-based geriatric assessment and care-coordination programs [4–15]. GEDIs aim to decrease adverse events such as falls, infection, and delirium. They address the persistent, unmet, health-related needs which are common among geriatric patients in the ED. [3, 5, 16] GEDIs are often evaluated with respect to their ability to optimize health care delivery, and in turn, reduce unnecessary repeat ED visits and re-hospitalization. Though these metrics are important measures of health services use, they are insufficient to understand the impact of GEDIs on patient-centered outcomes such as health-related quality of life (HRQoL).

HRQoL encompasses social, emotional, and physical well-being, and can be measured across multiple domains [17, 18]. It is often measured in chronic conditions like cancer, asthma, congestive heart failure, and frailty [19–22]. HRQoL is an important patient-centered outcome with implications for future ED use, mortality, and nursing home placement for geriatric patients [20, 23]. Understanding how geriatric patients value different domains of HRQoL before, during, and shortly after an ED visit is an important step in determining how to best measure HRQoL in this population. Better understanding older adults’ perspectives on HRQoL surrounding an ED visit may help to inform a patient-centered approach to the development, implementation, and evaluation of GEDIs.

The objective of this study was to describe which aspects of HRQoL are most valued by geriatric patients with a recent ED encounter and what expectations patients have for addressing or improving their HRQoL during an ED visit. A better understanding of how older adults view HRQoL in the context of an ED visit may inform the most appropriate measures of HRQoL to evaluate GEDIs in the future.

## Methods

### Study participants and setting

This was a qualitative focus group study of geriatric ED patients from an urban, academic ED with over 88,000 total annual visits (16,500 geriatric). Patients provided verbal consent at the time of the focus group as this study did not record and individually identifiable health information. The Institutional Review Board at Northwestern University approved all study procedures including the method of consent.

Patients were eligible if they were age 65 or older and discharged from the ED within 45 days prior to the start of recruitment. These patients were identified in March 2013 by the automated medical record electronic data warehouse (EDW) query for patients age > =65 years who were discharged from the ED (February – June 2013). Research assistants (RA) subsequently called identified patients for recruitment. Participants were screened at the time of the phone call for the following exclusion criteria: 1) non-English speaking, 2) uncorrected hearing impairment (as volunteered by patient or judged by RA). If interested, participants were assigned to one of the pre-established focus group times. Three patients participated in semi-structured pilot interviews performed to ensure participants had adequate understanding of the questions asked and to finalize the discussion guide prior to initiation of focus groups. Participants had no ongoing relationship with the moderators of the focus groups, though they may have been cared for in the ED by one of the moderators in the past.

### Data collection

Pilot interviews and focus groups were performed between March and June 2013 in the administrative offices of the Department of Emergency Medicine. Focus groups lasted 60 min and were audio-recorded. Participants were reimbursed for their time with a $25 gift card. Participants were read a verbal consent script on arrival to the focus group and the RA recorded their consent. For each focus group, one research team member (SD, DM, or KE) served as the moderator and a second researcher (SD, DM, or KE) wrote notes and assisted in summarizing the discussion. Moderators were all emergency medicine physicians with additional research training in qualitative methods. All interviews and focus groups were performed by 1 male and 1 female moderator who used a written focus group guide which had been developed by the research team and pilot tested with three participants. Phrasing was modified after pilot testing, but there were no significant changes to the content of the discussion questions. There were no repeat interviews or focus groups.

Participants were informed that the objective of the focus groups was to understand patient experiences in the ED of older adults and to better understand how care in the ED could impact quality of life. The moderator asked focus group participants to comment on their experience in the ED and how that experience related to their quality of life. Focus group discussion was facilitated using the guide developed during pilot interviews (Appendix). Participants were asked to think back to their visit in the ED and how that experience influenced their quality of life. They were asked how care in the ED could improve their physical health, mental health, and social health. In order to limit the impact of the focus group guide on participants’ responses, terms which may be unfamiliar to patients such as Quality of Life, Physical Health, Mental Health, and Social Health were not defined for participants. Rather the participants were asked what those terms meant to them and to discuss these concepts. They were then asked which impacts of ED treatment on quality of life were most important to them and why. Recruitment for additional focus groups stopped once researchers reviewed the transcripts and determined that sufficient saturation of themes was achieved.

### Data analysis

The pilot interviews and focus groups were de-identified and transcribed verbatim by a professional transcription service. Individual participants were not identified in the transcription. Transcriptions were not provided to participants.

Three coders (SD, DM, KE) experienced in qualitative methods used latent content and constant comparative methods to analyze the transcripts for emergent themes [24]. The coders independently reviewed the transcripts to familiarize themselves with the data. Coders then met as a group to compare notes and compile a preliminary list of emergent codes. Coders then alternated independent review of the transcripts with group meetings repeatedly until a final system of categories and overarching themes was developed. Any discrepancies were resolved through group discussion and consensus was achieved for all initial discrepancies. Codes, quotes, and themes were organized in Microsoft Excel. Participants were not informed of the findings of the analysis.

## Results

Three subjects participated in pilot interviews, and 31 subjects participated in one of 6 focus group sessions. The median age of participants was 70 (interquartile range = 67–74) and participants were 70% women. The most common discharge diagnoses were related to pain (29%), followed by infection (23%) (Table 1). Through consensus, 18 codes were generated, 5 codes pertained to the patients’ general experiences in the ED (Respect, Communication, ED physical environment, Time, and Need for dedicated geriatric EDs). 13 were related to HRQoL. The 13 codes related to HRQoL were categorized into themes.

**Table 1:** Participant discharge diagnoses

Diagnosis	*n* (total 31)
Pain	9
Infection	7
Fracture/Sprain	5
Laceration	4
Exacerbation of chronic illness	2
Miscellaneous	7

### Themes and codes

Five themes emerged as patients discussed their ED experience and the ED’s potential impact on their physical, mental, and social quality of life: (1) Functional recovery which included 3 codes – Physical health, Addressing pain and comfort, and Return to baseline. (2) Mental health and anxiety which included 4 codes: Availability of care, Mental health, Reassurance of recovery, and Understanding next steps. (3) *Interpersonal* effects of health on quality of life which included 3 codes: Social health, Interaction of physical health and social health, and Interaction of mental health and social health. (4) *Individual* experience of health and quality of life which included 2 codes: Changing quality of life priorities with age, Interaction of physical health and mental health. (5) Is the ED is the right place to discuss quality of life which was its own code (Table 2). These themes are described below with narrative examples of participant quotes.

**Table 2:** Additional Representative Quotes

Theme	Representative Quotes
Functional recovery	“I hurt, I want you to heal me.”
	“They gave me equipment so I could get around and maintain my lifestyle and gradually get back to where I was.”
	“To help you, help restore your vitality. And it’s true, if you don’t think that you’re going to be able to carry on with your daily activities and all the projects that you do, you think well you’re just really not participating fully in life anymore.”
	“You want them to be able to make sure that you can be as active as possible for as long as possible.”
Mental health and anxiety	“Otherwise, someone is going to worry themselves to death maybe over nothing. So, I realize patience in this case is a virtue, but sometimes I think when you’re so antsy and worried some reassuring words might help.”
	“Of course, as we get older we think of, oh my gosh, my friend died when she was only 60, she was only 58, this woman is 73, so we’re scared, we’re scared of crap, we really are and I don’t know what the answer is.”
	“Knowledge is really the most important thing. I would rather know, the suspense is worse than whatever news is going to be dished out, albeit you don’t want bad news but to wait around and to wonder and wonder and wonder.”
	“I was really very down temporarily about this. Thinking, oh, you know, this is never going to get better.”
Interpersonal effects of health on quality of life	“Well I think if you’re ill or injured to the point where you’re isolated, of course it affects your social relationships, particularly if you don’t have a live-in companion.”
	“But to be honest with you I was less concerned about that than I was about how whatever was going on with me would impact my wife, because she depends on me, okay? I have been married to the same woman for 40 years, my anniversary was this past Saturday. I was concerned about, you know, I can’t get sick now.”
	“I’m going to be 69 next month. I’m not 20, but I can, you know, if I want to go swim with my grandkids I can do that. If I want to bicycle with them I can do that, if I want to pick them up and toss them around I have the strength to do that.”
Individual experience of health and quality of life	“When you go there, it reminds you that you’re not a kid anymore, and everything isn’t self-healing and quickly done, and maybe in your imagination. That you in fact are not what you used to be. And the emergency room reminds you of that, and you don’t want to go back.”
	“What you experience on the outside affects you everywhere, it really does. It’s difficult to deal with sometimes because you tell yourself why is this happening?”
The ED is not the appropriate setting to address quality of life	“Would I expect an emergency room physician to be addressing those? They don’t even know me. They should be addressing the problem at hand and then send me off to my primary care physician for followup. That’s where it should be addressed.”
	“I don’t think it’s an emergency room issue. I think if you have a social issue, it’s an issue. I don’t think an emergency room impacts it in any way.”

### Functional recovery

Participants expressed a desire to improve physically to be able to return to their previous activities. Participants expressed that their quality of life was significantly influenced by *“being healthy”,* that they hoped to have their uncomfortable symptoms such as pain addressed and return to their symptomatic baseline, and that they desired a return to their baseline levels of activity and independence.

*“I want to return to the same degree of pain free mobility that I experienced before I fell.”*

### Mental health and anxiety

The emotional experience of needing and seeking emergency care was an anxiety provoking experience for many participants. Participants noted general concerns about their mental health. Some participants mentioned that their health made them sad or depressed. The uncertainty of not knowing what was happening, both in the process of care, and the ED diagnosis and prognosis led to stress and anxiety. Participants expressed anxiety while seeking reassurance that they would recover from their current medical problem and not be confined to a decreased quality of life. For some this anxiety continued after leaving the ED, participants expressed a desire to have a mechanism for following up on questions they had after leaving the ED. For some participants, knowing they had access to emergency care was reassuring.

*“I mean, when you’re at this stage of life, you know, you’re always afraid, like oh my God, is this thing going to be sort of permanent or something simple?”*

### Interpersonal effects of health on quality of life

Many patients identified having relationships, and the ability to interact with others as an important part of their *“normal life.”* Participants expressed concerns about social isolation and feeling alone both as a part of aging, and as a consequence of illness or injury. They also noted interactions between physical health and social health. The ability to function physically in their normal life helped to maintain their relationships and social health. Participants also identified an interaction between mental health and social health where poor mental health can lead to poor social health through isolation, but also how poor social health can lead to poor mental health.

*“If you don’t feel good, you don’t want to do anything. You don’t have a social life.”*

### Individual experience of health and quality of life

Participants noted that the ideas that are important to their quality of life are different now than they were when they were younger. They also noted how physical health impacts their mental health, and how mental health problems like anxiety and depression can affect physical health.

*“I was disturbed because, as I said, here I am trying to hobble around on this walker, my rib hurts, and I reached a point where I thought, “I can’t do this.” That was an emotional reaction, it wasn’t a physical assessment of the situation.”*

### Is the emergency department the right place to discuss quality of life?

Some participants questioned whether quality of life should be addressed at all in the ED. They mentioned that ED-based interventions must be limited, acknowledging that EDs *“can’t do everything,”* and that they primarily went to the emergency department to *“get healthy.”* There was some concern that efforts to improve HRQoL in the ED would be beyond the role of the ED.

*“I also think there are limits to what an emergency room visit can accomplish. There's got to be questions of priorities. I think if the emergency room sent me home with a talking teddy bear I'd be a little worried.”*

## Discussion

This study used a qualitative approach to understand which aspects of HRQoL are important to geriatric patients when they seek care in the ED. We found that geriatric patients had concerns about returning to their baseline functional status, they demonstrated anxiety and stress related to uncertainty around their health, and they identified connections between their physical, mental, and social health that are important to their overall quality of life.

Previous studies have shown that HRQoL worsens for older adults during an acute illness, gradually improves over time, but often never returns to baseline levels [25]. Additionally, low HRQoL has been associated with need for assistance and mortality [26, 27]. However, to our knowledge, this is the first study to evaluate how geriatric patients conceptualize their HRQoL around the time of an ED visit.

Our question guide centered on the three major domains of HRQoL and, not surprisingly, several of the emergent themes paralleled those domains. However, the depth of the comments within the themes may be helpful in designing future interventions to make geriatric ED care more patient centered. The first theme, focusing on functional recovery, aligns with the physical domain of HRQoL. Although one can generally assume that most patients (irrespective of age) are seeking recovery, the quotes within this theme demonstrate that geriatric patients are very focused on *function*, mentioning their activities of daily living, “mobility” and “vitality.” A heightened awareness of patients’ concerns regarding functional recovery can facilitate discussions regarding rehabilitation and care coordination, as well as the engagement of resources to help patients achieve these goals. In particular, such discussions can lead simple interventions or modifications in the home environment that can ensure patients maintain mobility and function in the setting of acute illness or injury.

Whereas physical recovery is often addressed during ED visits, acknowledging the significant interaction between physical health and mental or social health likely occurs less frequently. Many statements seen in the second theme (“mental health and anxiety”) focus on prognosis and patients seeking an answer to the question of “is this going to be permanent.” Frequently that answer is not available at the time of the ED visit as recent study found that up to 37% of ED encounters end without a definitive pathologic diagnosis [28]. However, understanding patients’ anxiety about their diagnosis and prognosis may help clinicians to discuss the longer-term implications or what future testing is needed to make a determination. Several recent studies examining patients’ rationale for ED return visits also found that fear and uncertainty about the unknown was a driving factor, suggesting this is not a concern unique to geriatric patients [29, 30].

Addressing the mental aspects of HRQoL, begins with clinician awareness and inquiry. Patients described emotions, experiences and concerns that were related to their acute illness or injury. These issues were not acknowledged or addressed in the ED – and interestingly, patients generally did not expect this. At the same time, patients did express a desire for more reassurance and greater acknowledgement as a ‘whole person’. These findings identify an unmet patient need with respect to patients’ HRQoL, but also raise questions regarding how these needs can best be addressed. ED provider education should heighten awareness regarding the particular significance of mental health issues for HRQoL among geriatric patients and reinforce the importance of talking to patients about this topic. At the same time, it is clear that patients don’t expect us to resolve these challenges in the ED and our role as emergency physicians should be to acknowledge them and, in turn, facilitate interventions and support after discharge.

Interestingly, aging and mortality were not themes that emerged from our analysis. There are many possible reasons these important topics were not discussed. Other research has indicated that death and dying are seldom discussed, even in nursing homes where many older adults die [31]. In the ED, clinicians often feel uncomfortable discussing death and introducing the idea of palliative care [32]. Additionally, the framework of the focus groups which centered on HRQoL and were conducted by emergency physicians may have influenced participants responses regarding aging and death. Previous research of patients with life threating conditions has indicated that older adults begin to turn inwards to come to peace with the past, the present and approaching death when they are trapped by health complaints [33]. In our study, selection of patients who are discharged may have influenced their comfort with discussing death and aging. The included patients may not have felt “trapped by health complaints” as participants in previous studies have. Including older adults who were hospitalized with more severe injuries or illnesses may have led to additional discussion on these important topics.

In addition to informing clinical care of geriatric patients in the ED, the results of our study may be most applicable in HRQoL evaluations of GEDIs in the future. Currently recommended evaluation of GEDIs include reporting metrics such as admission rates, repeat ED visits, and readmissions and disease specific process measures such as use of urinary catheters and restraints [34, 35]. Though these are important health system measures, a more patient-centered approach, supported by the National Institute on Aging (NIA) and Agency for Healthcare Research and Quality (AHRQ), would incorporate patient centered outcomes such as HRQoL [36]. This approach may be particularly important for GEDIs because HRQoL is predictive of mortality and nursing home placement for older adults in the ED. [23]

A potential tool for evaluating HRQoL in GEDIs is the Patient Reported Outcomes Measurement Information System (PROMIS), a system of highly reliable, validated and precise measures of patient-reported HRQoL [18, 37]. PROMIS has multiple pre-existing “domains” that can be tailored using computer adaptive testing. The results of this study show a good link between available PROMIS domains (e.g. physical function, anxiety, satisfaction with social roles) and spontaneously generated themes from patients. The *domain specific* measures available within PROMIS could thereby allow for longitudinal analysis of HRQoL after an ED visit and comparison to age-specific national benchmarks across multiple domains of HRQoL [38]. Future studies using measures such as PROMIS to evaluate changes in specific domains of HRQoL during and after an ED visit are warranted. If these measures are reliably collected after during and after an ED visit, they could be considered for use in evaluating GEDIs in the future.

### Limitations

The focus group guide was carefully designed to avoid leading questions. However, HRQoL was not a term that many participants were familiar with and some introduction of the topic was necessary. Though this topic was introduced by posing questions to participants rather than providing a definition, it is possible that some participant responses were steered by the format and question guide. Additionally, focus group participants may be more likely to give socially desirable responses and to please the moderators. Despite this possible limitation, participants were unafraid to question whether the ED is the right place to discuss HRQoL, though they may have had additional objections which were not mentioned. All participants were discharged from the ED and asked to return for focus groups, which likely introduced some selection bias. Participants were likely healthier with better access to transportation than the overall population of geriatric patients in our ED. However, even among this likely healthier cohort, significant concerns about HRQoL arose which were recalled weeks after an ED visit. From our data patient comorbidities are unknown. Patients with chronic health conditions such as cancer or severe congestive heart failure may have additional HRQoL concerns that were not brought up in these focus groups of patients discharged from the ED. Additionally, this was a single site study and the results may not be generalizable to geriatric patients in other emergency departments. Despite these limitations, focus groups are an important qualitative method to evaluate new concepts such as how older adults conceptualize HRQoL at the time of an ED visit, how they would prioritize care in the ED, and how care in the ED can influence HRQoL.

## Conclusion

The geriatric patients discharged from the ED in this study discussed how their illness or injury not only caused physical symptoms, but also impacted relationships and mental health. Although some patients questioned if the ED was the proper venue to address HRQoL, they expressed feelings of anxiety around the time of their ED visit related to uncertainty, they desired functional recovery, and identified both interpersonal effects of health on quality of life mediated by social health, and an individual experience of health and quality of life mediated by mental health. Clinicians caring for older adults may consider framing their discussions to relieve anxiety when possible, and to address the prospects of a functional recovery. As GEDIs, including dedicated GEDs continue to proliferate, measurement of priority domains of HRQoL for older adults’ in the ED should be part of the evaluation of these innovative programs.

## Data Availability

The datasets generated an analyzed during the current study are not publicly available due to participant privacy but may be available from the corresponding author upon reasonable request after consultation with the Northwestern University Institutional Review Board.

## Appendix

**Table 3:** Focus Group Guide

Questions for the Group	
Please take a few minutes and “think back” to your visit to the ED. Let’s start by thinking about your experience in the emergency department. What was it like being a patient in the emergency department? ● Was that how it went for everyone else? ● What could be improved about the experience of being a patient in the ED?	
OK. Now let’s think about how your experience in the emergency department could help your quality of life. What does the term “quality of life” mean to you? ● How does your health affect your quality of life?	
What does the term physical health mean to you? ● How does your physical health affect your quality of life? ● What can the emergency department do to improve your physical health?	
What does the term mental health mean to you? ● How does your mental health affect your quality of life? ● What can the emergency department do to improve your mental health?	
What does the term social health mean to you? ● What can the emergency department do to improve your social health?	
Of all the things we discussed about how your evaluation and treatment in the ED can affect your quality of life and health, what is the most important? ● What makes it important?	
*Moderator give a short summary of the key questions and big ideas that emerged from the discussion*	
How well does that summary capture what was said here? ● Is there anything that we should have talked about but didn’t?	

## Abbreviations

ED: Emergency Department
EDW: Enterprise Data Warehouse
GED: Geriatric Emergency Department
GEDI: Geriatric Emergency Department Innovation
HRQOL: Health Related Quality of Life
